# Reported infant safety information and perinatal service engagement among birth partners and co-parents. An exploratory cross-sectional survey at a large UK teaching hospital

**DOI:** 10.3389/fped.2026.1884421

**Published:** 2026-10-13

**Authors:** Olufisayo Olakotan, Modther Syed, Ahmed Abudraz, Camilla Benouali, Muhammad Ali, Nicole Chua, Su'ad Takar, Theubo Miller, William Dodwell, Fatima Tiamiyu Mogaji, Sanaa Rashwan, Elmansi Ahmed, Priya Sivakumaran, Megan O'Kerr, Samuel Atiku, Manjiri Khare, Thillagavathie Pillay

**Affiliations:** 1 Department of Research and Development, The Royal Wolverhampton NHS Trust, Wolverhampton, United Kingdom; 2 Department of Neonatology, University Hospitals Leicester NHS Trust, Leicester, United Kingdom; 3 Department of Obstetrics, University Hospitals Leicester NHS Trust, Leicester, United Kingdom; 4 Research Service, Aston University, Birmingham, United Kingdom; 5 Department of Population Health Sciences, University of Leicester, Leicester, United Kingdom; 6 Faculty of Science and Education, University of Wolverhampton, Wolverhampton, United Kingdom

**Keywords:** birth partner, co-parent, health inequalities, perinatal education, self-reported knowledge, STROBE

## Abstract

**Background:**

Infant mortality in the United Kingdom is higher in areas of socioeconomic deprivation and within several minority ethnic groups. Preventive strategies rely in part on safety information reaching families during the perinatal period. The extent to which birth partners and co-parents report exposure to that information is not well characterised.

**Objective:**

To describe self-reported exposure to selected infant safety topics and reported engagement with perinatal services among birth partners and co-parents attending one large UK teaching hospital, and to explore variation across respondent profiles defined by ethnicity and parity.

**Methods:**

An exploratory cross-sectional survey of 73 birth partners and co-parents was conducted at University Hospitals Leicester NHS Trust between October 2022 and July 2023. Eight self-reported knowledge or awareness items and six self-reported engagement items were collected. Item-level proportions were summarised with Wilson 95 percent confidence intervals. Group comparisons used Fisher's exact test with Haldane-corrected prevalence ratios and Benjamini–Hochberg adjustment applied separately within each demographic comparison. Composite Knowledge Proportion Scores and Engagement Proportion Scores were computed for respondents with at least half of the relevant items answered. Internal consistency was estimated with the Kuder-Richardson 20 coefficient, association with Spearman's rank correlation, and group differences with the Mann–Whitney *U* test. Reporting follows the STROBE statement for cross-sectional studies.

**Results:**

Reported exposure was highest for smoking risk (69.9 percent, 95 percent CI 58.6–79.2) and lowest for basic bystander life support (17.8 percent, 95 percent CI 10.7–28.1). Engagement was highest for NHS website use and post-birth breastfeeding support (both 61.6 percent, 95 percent CI 50.2–71.9) and lowest for antenatal classes (24.7 percent, 95 percent CI 16.2–35.6). Composite scales showed high internal consistency (KR-20 of 0.94 for knowledge, 0.93 for engagement). The two composite scores were strongly correlated (Spearman's *ρ* = 0.965, *p* < 0.001). Ethnicity and parity were completely confounded in the usable sample, with all first-time parents identifying as White British and all respondents in the combined Other category identifying as experienced parents. Observed differences between these respondent profiles cannot be attributed independently to ethnicity or parity.

**Conclusions:**

In this exploratory single-site sample, self-reported exposure to practical infant emergency-care topics was low. Substantial differences were observed between two contrasting respondent profiles, although the confounded design and small convenience sample do not support causal claims about ethnic inequities or infant mortality risk reduction. The findings are hypothesis-generating and support the case for larger, more diverse studies designed to separate the contributions of ethnicity, parity, and other social determinants.

## Introduction

Birth partners and co-parents, most often the infant's father or the mother's chosen support person, occupy a functional role in perinatal and infant care that extends well beyond the labour ward. Their role includes shared caregiving, decision-making, and support for maternal recovery (1, 2). Intervention studies suggest that partner-inclusive programmes may improve breastfeeding uptake and maternal mental health outcomes (3, 4). Qualitative work indicates that many partners still experience maternity care as oriented primarily toward mothers and report feeling peripheral to clinical interactions (1, 2, 5). Reports of exclusion are more frequently described by partners from ethnically diverse backgrounds (1, 5, 6). The institutional gap is visible. Partner-facing safety education appears uneven in design and unevenly distributed in reach.

Infant mortality in the United Kingdom patterns along ethnicity and socioeconomic deprivation, with several observational and mortality-review studies reporting persistent gaps (7–15). Preventable risks such as unsafe sleep, smoke exposure, and delayed response to infant illness recur across those reviews (8, 11, 13, 14). These risks demand practical family knowledge, and in some scenarios rapid action. A plausible pathway links partner exposure to safety information with family preparedness and caregiving practices, and those practices may in turn influence safety-related outcomes. Opportunities to receive, interpret, and act on information may themselves be shaped by inclusion in clinical contacts, previous parenting experience, socioeconomic constraints on attendance, language and health literacy, digital access, and the accessibility of services (1, 2, 5, 6, 15). These determinants may cluster with ethnicity and parity rather than operate as independent causes, which is especially important in the present sample where ethnicity and parity are completely confounded. The present survey measures only self-reported exposure and engagement. It does not measure caregiving behaviour or infant outcomes, so the downstream links in this conceptual pathway are not tested.

The literature on birth partner and father involvement in perinatal education is now substantial (1–6, 16, 17). Less is known about a specific subquestion, namely the extent to which birth partners themselves report exposure to defined infant safety topics, including practical emergency-care skills such as choking response, basic life support, and first aid, and how that exposure varies across respondent groups defined by ethnicity and parity. This study addresses that narrower descriptive question at one large UK teaching hospital. It examines self-reported exposure to eight safety topics, self-reported engagement with six perinatal services and information resources, variation across two contrasting respondent profiles, and the internal consistency of two composite indices. The study is exploratory and hypothesis-generating. It was not designed or powered to test causal claims about ethnic inequities or about infant mortality risk reduction.

## Methods

### Study design and setting

A single-centre exploratory cross-sectional survey was conducted at University Hospitals Leicester NHS Trust, which delivers approximately 10,000 births per year. The work formed part of a service evaluation. The recruitment period ran from 1 October 2022 to 31 July 2023. This 10-month window was set to align with the funded service-evaluation cycle. No structural changes to the antenatal education programme, midwifery staffing, or the Trust's birth-partner information leaflets are known to have occurred within this period. Reporting follows the STROBE statement for cross-sectional observational studies (18). The completed checklist is provided as Supplementary Material/Appendix A.

### Participants and recruitment

Eligible participants were birth partners or nominated co-parents of pregnant women or of women who had given birth within the prior 4 weeks and who were attending antenatal or postnatal appointments at the Trust during the recruitment window. Convenience sampling was used. Research team members approached partners in antenatal outpatient clinics on weekday mornings and afternoons, and in postnatal ward areas on weekdays. Recruitment was not consecutive. Recruitment was not attempted at weekends, in the evening, in the delivery suite, or in the neonatal unit. Partners with insufficient English proficiency to complete the questionnaire without assistance were not enrolled, as translated versions of the instrument and formal interpreter services were not available.

Of partners approached during the recruitment window, 73 completed the questionnaire. A record of the number approached, the number who declined, and the reasons for declining was not systematically maintained, so a formal response rate cannot be reported. The 73 respondents formed a convenience sample. The sample size was not derived from a formal power calculation. It reflects the number of birth partners approached, eligible, and willing to complete the questionnaire within the funded ten-month window.

### Recruitment period, antenatal and postnatal strata

Forty respondents were surveyed during their partner's antenatal period. Thirty-three were surveyed during the postnatal period. Some outcomes are intrinsically shaped by where a respondent sits on the perinatal pathway. Post-birth breastfeeding support may not be applicable to antenatal respondents. Item-level results should be read as time-conditional on recruitment period. Cross-tabulations of recruitment period against ethnicity and parity are provided in Supplementary Table S1. Sensitivity analyses stratifying the item-level results by recruitment period are provided in Supplementary Table S2. The stated median gestational age at survey was 36 weeks. For postnatal respondents, this refers to the pregnancy gestational age at the time of the birth, not the interval since birth.

### Survey content and measures

The questionnaire was developed by the study team drawing on national guidance for perinatal education and on Trust-level infant-safety information sheets (17). It was reviewed for face content by clinical members of the study team, including a consultant neonatologist and a senior midwife. It was not formally piloted and was not adapted from a previously validated instrument. The face review was pragmatic and should not be interpreted as formal instrument validation. All measures capture self-reported exposure to information or self-reported contact with services rather than objectively tested knowledge or observed behaviour. The eight knowledge or awareness items covered benefits of breastfeeding, basic bystander life support, dealing with a choking infant, recognising signs of illness in a baby, risks of smoking, risks associated with shaking or coping with a crying baby, safe sleep practice, and first aid. The six engagement items covered access to mental health support, attendance or intended attendance at antenatal classes, use of the NHS pregnancy or infant-health website, awareness of Lullaby Trust support, breastfeeding support received before birth, and breastfeeding support received after birth. Most items used a three-option response of Yes, No, or Unsure, with the antenatal-classes item fielded as a binary Yes or No question. Items left blank were coded as unanswered.

### Terminology

The terms knowledge, awareness, and exposure are used interchangeably to denote a respondent's self-report of having received information on a given topic. Engagement denotes self-reported contact with a service, resource, or organised information source. Birth partner and co-parent denote the mother's nominated support person, most commonly the infant's father, husband, or fiancé. Data on partner sex were not collected.

### Composite indices

Two composite indices were derived. The Knowledge Proportion Score (KPS) was calculated as the proportion of the eight knowledge items answered Yes, multiplied by one hundred. The Engagement Proportion Score (EPS) was calculated in the same way using the six engagement items. Each item was weighted equally within its index, so one affirmative item contributes 12.5 percentage points to KPS and approximately 16.7 percentage points to EPS when all items are answered. Equal weighting was a pragmatic descriptive choice because there was no empirical basis for differential weights, and it does not imply that the items are equivalent in clinical importance. Composite scores were computed only for respondents with at least half of the relevant items answered (four of eight for KPS, three of six for EPS). Higher scores denote greater reported exposure and greater reported engagement, respectively. Internal consistency was estimated with the Kuder-Richardson 20 coefficient, the analogue of Cronbach's alpha for binary indicators (19). The KPS and EPS are descriptive summary indices. They were not derived from prior instrument validation and their psychometric properties were not established through formal pilot testing, factor analysis, or comparison with an external criterion. The association between KPS and EPS was estimated with Spearman's rank correlation coefficient (*ρ*).

### Data preparation and recoding

Ethnicity was self-described. Responses of White British were coded as White British. All remaining ethnic self-descriptions were combined into a single Other category, since no other single ethnic self-description reached a cell count sufficient for stable subgroup estimation. The resulting Other category is heterogeneous. It includes respondents self-describing as Romanian, Kurdish, Chinese, African, Indian, Pakistani, Bengali, Albanian, Nigerian, and Polish. The category combines national, regional, and pan-ethnic identifiers and should not be interpreted as representing a coherent ethnic community. Parity was coded as First-time for responses indicating no previous children and as Experienced for responses indicating one or more previous children. All knowledge and engagement items were converted to binary indicators. Yes was coded as one, No as zero. Treatment of Unsure varied between the primary and sensitivity analyses, as described below. Items left blank were coded as missing and excluded from the denominator for that item. The raw distribution of Yes, No, Unsure, and missing responses for each item is provided in Supplementary Table S3.

### Statistical analysis

All analyses were carried out in Python 3 using pandas, NumPy, scipy.stats, and Matplotlib; Wilson confidence intervals and Benjamini–Hochberg adjustment were implemented directly in the analysis script. The full analytical script is provided as Supplementary Material A. The analytical plan proceeded through five stages. The first stage produced descriptive frequencies for each item, with Wilson 95 percent confidence intervals around each Yes proportion. Wilson intervals were chosen because they remain stable when proportions approach zero or one, conditions that arise repeatedly in this dataset (20, 21). The second stage tested group differences at the item level using Fisher's exact test, chosen because cell counts were small and several were zero. Effect sizes were reported as Haldane-corrected prevalence ratios with 95 percent confidence intervals. The ratios were calculated as White British divided by Other for ethnicity and First-time divided by Experienced for parity, making Other and Experienced the respective reference categories. Ratios above one therefore indicate a higher observed prevalence in White British or First-time respondents relative to the corresponding reference group. Prevalence ratios are appropriate here because the design is cross-sectional. A 0.5 continuity correction was applied to all cells when any cell was zero, so that the log-transform confidence interval remained defined under complete separation (22, 23).

The third stage addressed multiple testing. Fourteen item-level Fisher's exact *p*-values were adjusted with the Benjamini–Hochberg false discovery rate procedure (24). The adjustment was applied separately within each demographic comparison, namely fourteen tests for ethnicity and fourteen tests for parity, rather than jointly across all twenty-eight tests. Both unadjusted *p*-values and adjusted *q*-values are presented. The fourth stage examined internal consistency and cross-scale association for the composite indices. KR-20 was computed for each item set. Spearman's *ρ* quantified the association between KPS and EPS overall, and separately within each respondent profile where sample size permitted. The fifth stage compared composite scores between groups using the Mann–Whitney *U* test, since both KPS and EPS were skewed with substantial floor effects.

### Sensitivity analyses

Three sensitivity analyses were specified for the revised analysis. First, the primary analysis treated Unsure responses as equivalent to No, on the reasoning that uncertainty in a safety-critical context may function in practice much as not knowing. A sensitivity analysis recoded Unsure as missing and excluded those responses from denominators. Second, item-level results were stratified by recruitment period (antenatal vs. postnatal). Fisher's exact tests for the 14 item-level recruitment-period comparisons were adjusted with Benjamini–Hochberg correction, and KPS and EPS were compared between recruitment periods using Mann–Whitney *U* tests. Third, item-level results were tabulated separately for the demographic respondent groups to allow the reader to inspect the observed pattern alongside the complete ethnicity-parity confounding. The substantive direction and magnitude of the primary findings were consistent across these checks.

### Handling of missing data

An available-case approach was used throughout. For any given item-level comparison, only respondents with valid data on the item were retained. Composite scores were computed only for respondents with at least half of the relevant items answered. Item-level missingness was low across the questionnaire. Only one item (first aid) had any missing responses, at 1 of 73 (1.4 percent). All other items had complete data. Full item-level missingness counts are provided in Supplementary Table S3. Multiple imputation was not used, since the missingness mechanism could not be modelled with the available data and the proportion of missing responses was low.

### Variables not measured

The questionnaire did not collect individual-level information on educational attainment, employment status, occupation, area-level deprivation, migration history, English language proficiency, digital access, or health literacy. These variables are plausibly related both to access to perinatal information and to engagement with services and could account for part of the between-group variation observed in this study. Their absence is a substantive limitation of the analysis. Respondent age and relationship to the mother were collected as descriptive characteristics but were not incorporated into the inferential comparisons, because the small sample would not support meaningful stratification beyond the two-way ethnicity and parity contrasts already presented.

## Results

### Sample characteristics

Of the 73 birth partners and co-parents who completed the questionnaire, 40 were surveyed during their partner's antenatal period and 33 during the postnatal period. Median gestational age at the time of the pregnancy under discussion was 36 weeks in both strata. Respondents were predominantly the infant's partner, fiancé, or husband (*n* = 52, 71.2 percent). Sixty-six respondents reported that the baby would be cared for mainly by family members, and twelve planned to use nursery services during the first year of life. Three did not respond to this item. Full sample characteristics with Wilson 95 percent confidence intervals for each item-level proportion are presented in Table 1. Sample characteristics stratified by recruitment period are provided in Supplementary Table S1.

**Table 1:** Characteristics of the 73 birth partners and co-parents surveyed, with Wilson 95 percent confidence intervals.

Characteristic	Category	*n* (%) [95% CI]
Recruitment period	Antenatal	40 (54.8) [43.4, 65.7]
	Postnatal	33 (45.2) [34.3, 56.6]
Ethnicity	White British	45 (61.6) [50.2, 71.9]
	Other (10 self-described identities)	28 (38.4) [28.1, 49.8]
Parity	First-time parent	36 (49.3) [38.2, 60.5]
	Experienced (1 or more previous children)	37 (50.7) [39.5, 61.8]
Age range (years)	16–21	2 (2.7) [0.8, 9.5]
	22–30	24 (32.9) [23.2, 44.3]
	31–40	41 (56.2) [44.8, 67.0]
	Over 40	6 (8.2) [3.8, 16.8]
Relationship to mother	Husband, fiancé, or partner	52 (71.2) [60.0, 80.3]
	Mother	1 (1.4) [0.2, 7.4]
	Aunt	1 (1.4) [0.2, 7.4]
	Not declared	19 (26.0) [17.3, 37.1]

The Other ethnicity category is heterogeneous and combines respondents self-describing as Romanian, Kurdish, Chinese, African, Indian, Pakistani, Bengali, Albanian, Nigerian, and Polish. Wilson 95 percent confidence intervals are shown.

### Item-level self-reported exposure and engagement

Reported exposure varied across the eight knowledge topics. Awareness of smoking risk was reported most frequently (69.9 percent, 95 percent CI 58.6–79.2). Bystander basic life support was reported least often (17.8 percent, 95 percent CI 10.7–28.1). Reported exposure to information about first aid, choking response, and recognising signs of illness in an infant sat in the lower half of the distribution. Engagement items showed a related, although not identical, distribution. NHS website use and post-birth breastfeeding support were each reported by 61.6 percent of respondents (95 percent CI 50.2–71.9). Antenatal class attendance or intended attendance was the lowest, at 24.7 percent (95 percent CI 16.2–35.6). The combined item-level distribution is shown in Figure 1. Stratification by recruitment period is reported in Supplementary Table S2. No item-level antenatal-postnatal comparison remained statistically significant after Benjamini–Hochberg correction, with the smallest raw *p*-value observed for post-birth breastfeeding support (*p* = 0.0308, *q* = 0.353). Composite KPS and EPS also showed no statistical evidence of a recruitment-period difference (Mann–Whitney *p* = 0.730 and *p* = 0.204, respectively). The largest descriptive between-strata differences nevertheless involved outcomes that are intrinsically time-conditional in the perinatal pathway, particularly post-birth breastfeeding support (Table 1b).

**Figure 1:**
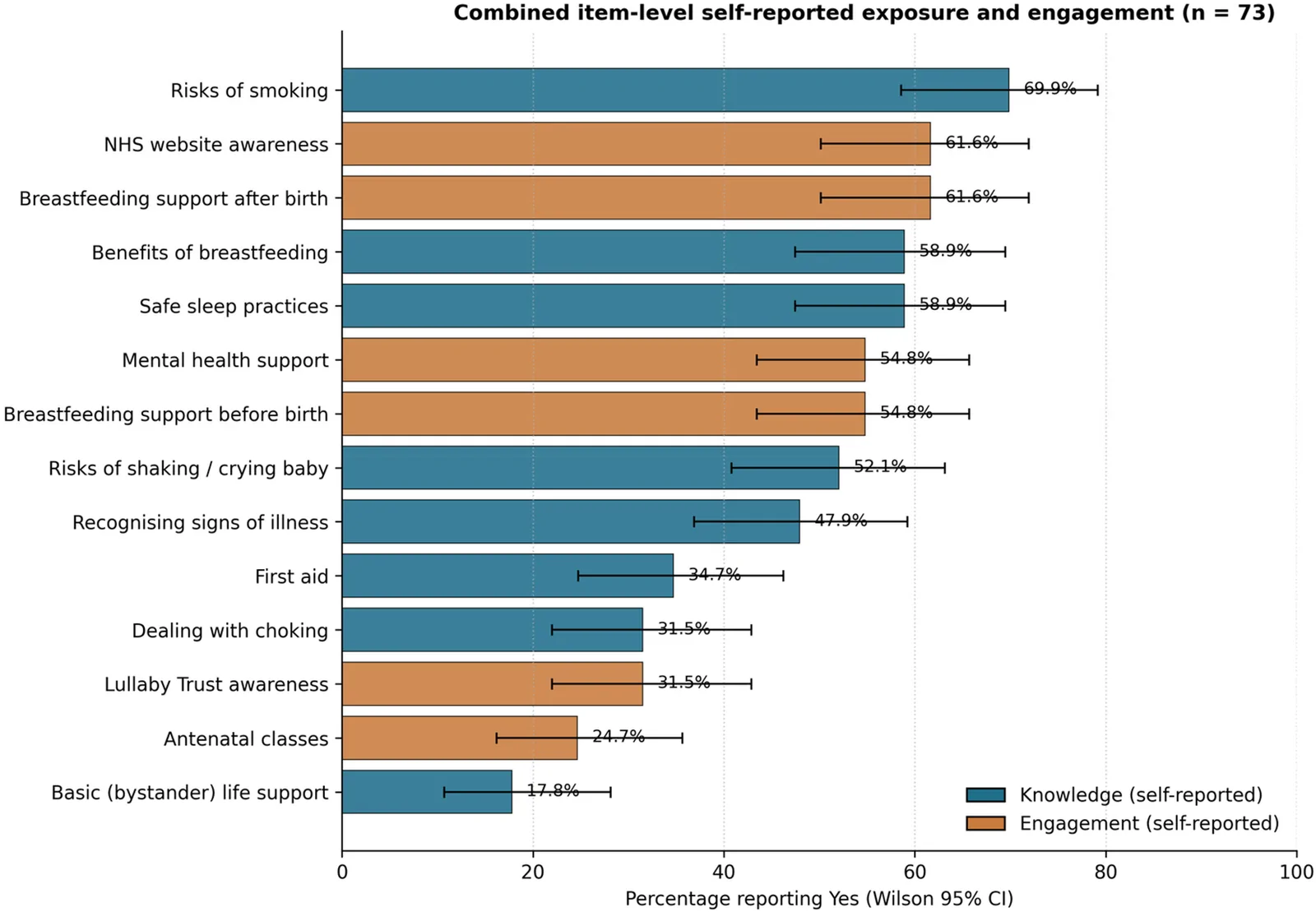
Combined item-level self-reported frequencies for the eight knowledge items and six engagement items reported by 73 birth partners and co-parents. Yes responses are shown as percentages on the horizontal axis, ranked from highest to lowest. Knowledge items are shaded in one colour and engagement items in a second. Whiskers indicate Wilson 95 percent confidence intervals.

**Table 1b:** Item-level self-reported knowledge and engagement, with Wilson 95 percent confidence intervals (*n* = 73).

Item	Yes *n*/*N*	% [95% CI]
Knowledge or awareness (self-reported)		
Benefits of breastfeeding	43/73	58.9 [47.4, 69.5]
Basic (bystander) life support	13/73	17.8 [10.7, 28.1]
Dealing with choking	23/73	31.5 [22.0, 42.9]
Recognising signs of illness in an infant	35/73	47.9 [36.9, 59.2]
Risks of smoking	51/73	69.9 [58.6, 79.2]
Risks of shaking/coping with a crying baby	38/73	52.1 [40.8, 63.1]
Safe sleep practices	43/73	58.9 [47.4, 69.5]
First aid	25/72	34.7 [24.8, 46.2]
Engagement with services and information sources (self-reported)		
Mental health support	40/73	54.8 [43.4, 65.7]
Attended or intended to attend antenatal classes	18/73	24.7 [16.2, 35.6]
NHS website awareness	45/73	61.6 [50.2, 71.9]
Lullaby Trust support awareness	23/73	31.5 [22.0, 42.9]
Breastfeeding support before birth	40/73	54.8 [43.4, 65.7]
Breastfeeding support after birth	45/73	61.6 [50.2, 71.9]

All items are self-reported by the birth partner or co-parent. First aid *n* = 72 because of one unanswered response. All other items *n* = 73. The raw distribution of Yes, No, Unsure, and missing responses for each item is provided in Supplementary Table S3.

### Group comparisons by ethnicity

Item-level comparisons between the White British and Other respondent profiles are presented in Table 2. The White British group reported the higher proportion on every item. Between-group differences were large in absolute terms. For example, antenatal class attendance or intention to attend was reported by 18 of 45 White British respondents (40.0 percent) and by none of the 28 respondents in the Other group (0.0 percent), an absolute difference of 40.0 percentage points. Smoking risk awareness was reported by all 45 White British respondents (100 percent) and by 6 of 28 Other respondents (21.4 percent), an absolute difference of 78.6 percentage points. Haldane-corrected prevalence ratios were large and accompanied by very wide confidence intervals, reflecting the small Other group and the frequent zero cells in that group. Absolute differences between the two respondent profiles, reported alongside the prevalence ratios in Table 2, are the more informative summary of magnitude given the wide ratio intervals.

**Table 2:** Item-level self-reported knowledge and engagement, comparison between ethnicity-defined respondent groups.

Item	White British % (*n*/*N*) [95% CI]	Other % (*n*/*N*) [95% CI]	Absolute difference (pp)	Prevalence ratio (95% CI), *q*
Knowledge				
Benefits of breastfeeding	88.9 (40/45) [77, 95]	10.7 (3/28) [4, 27]	+78.2	8.30 (2.83, 24.29), *q* < 0.001
Basic life support	28.9 (13/45) [18, 43]	0.0 (0/28) [0, 12]	+28.9	17.02 (1.05, 275.53), *q* = 0.0011
Choking	51.1 (23/45) [37, 65]	0.0 (0/28) [0, 12]	+51.1	29.63 (1.87, 469.27), *q* < 0.001
Recognising an ill infant	77.8 (35/45) [64, 87]	0.0 (0/28) [0, 12]	+77.8	44.76 (2.85, 701.83), *q* < 0.001
Smoking risk	100.0 (45/45) [92, 100]	21.4 (6/28) [10, 40]	+78.6	4.41 (2.24, 8.69), *q* < 0.001
Shaken-baby risk	82.2 (37/45) [69, 91]	3.6 (1/28) [1, 18]	+78.7	23.02 (3.34, 158.53), *q* < 0.001
Safe sleep	95.6 (43/45) [85, 99]	0.0 (0/28) [0, 12]	+95.6	54.85 (3.51, 856.88), *q* < 0.001
First aid	55.6 (25/45) [41, 69]	0.0 (0/27) [0, 12]	+55.6	31.04 (1.97, 490.09), *q* < 0.001
Engagement				
Mental health support	84.4 (38/45) [71, 92]	7.1 (2/28) [2, 23]	+77.3	11.82 (3.09, 45.21), *q* < 0.001
Antenatal classes	40.0 (18/45) [27, 55]	0.0 (0/28) [0, 12]	+40.0	23.33 (1.46, 372.39), *q* < 0.001
NHS website	97.8 (44/45) [88, 100]	3.6 (1/28) [1, 18]	+94.2	27.38 (3.99, 187.71), *q* < 0.001
Lullaby Trust	51.1 (23/45) [37, 65]	0.0 (0/28) [0, 12]	+51.1	29.63 (1.87, 469.27), *q* < 0.001
Breastfeeding support before birth	86.7 (39/45) [74, 94]	3.6 (1/28) [1, 18]	+83.1	24.27 (3.53, 166.87), *q* < 0.001
Breastfeeding support after birth	88.9 (40/45) [77, 95]	17.9 (5/28) [8, 36]	+71.0	4.98 (2.23, 11.09), *q* < 0.001

Prevalence-ratio reference category is Other. All items are self-reported by the birth partner or co-parent. Wilson 95 percent confidence intervals are shown for each proportion. Prevalence ratios are Haldane-corrected to allow estimation in the presence of zero cells and are calculated as White British divided by Other. pp, percentage points. *q* is the Benjamini–Hochberg false-discovery-rate adjusted *p*-value from Fisher's exact tests, applied separately across the 14 ethnicity comparisons.

### Group comparisons by parity

Item-level comparisons between First-time and Experienced respondents are presented in Table 3. The pattern mirrors the ethnicity comparison. First-time parents reported higher proportions on every knowledge and engagement item. The two comparisons cannot be interpreted independently of one another. Ethnicity and parity are completely confounded in the usable sample. All 36 first-time parents identified as White British, and all 28 Other respondents identified as experienced parents. The remaining nine White British respondents were experienced parents. The point estimates and confidence intervals in Tables 2, 3 should be read as describing two contrasting respondent profiles in this sample, rather than as estimates of separable demographic effects. This constrains interpretation throughout.

**Table 3:** Item-level self-reported knowledge and engagement, comparison between parity-defined respondent groups.

Item	First-time % (*n*/*N*) [95% CI]	Experienced % (*n*/*N*) [95% CI]	Absolute difference (pp)	Prevalence ratio (95% CI), *q*
Knowledge				
Benefits of breastfeeding	100.0 (36/36) [90, 100]	18.9 (7/37) [9, 34]	+81.1	5.00 (2.63, 9.50), *q* < 0.001
Basic life support	36.1 (13/36) [22, 53]	0.0 (0/37) [0, 9]	+36.1	27.73 (1.71, 449.75), *q* < 0.001
Choking	63.9 (23/36) [47, 78]	0.0 (0/37) [0, 9]	+63.9	48.27 (3.04, 766.00), *q* < 0.001
Recognising an ill infant	91.7 (33/36) [78, 97]	5.4 (2/37) [1, 18]	+86.3	16.96 (4.39, 65.52), *q* < 0.001
Smoking risk	100.0 (36/36) [90, 100]	40.5 (15/37) [27, 56]	+59.5	2.42 (1.65, 3.55), *q* < 0.001
Shaken-baby risk	97.2 (35/36) [86, 100]	8.1 (3/37) [3, 21]	+89.1	11.99 (4.05, 35.53), *q* < 0.001
Safe sleep	100.0 (36/36) [90, 100]	18.9 (7/37) [9, 34]	+81.1	5.00 (2.63, 9.50), *q* < 0.001
First aid	69.4 (25/36) [53, 82]	0.0 (0/36) [0, 10]	+69.4	51.00 (3.22, 807.05), *q* < 0.001
Engagement				
Mental health support	100.0 (36/36) [90, 100]	10.8 (4/37) [4, 25]	+89.2	8.33 (3.50, 19.85), *q* < 0.001
Antenatal classes	50.0 (18/36) [34, 66]	0.0 (0/37) [0, 9]	+50.0	38.00 (2.38, 607.86), *q* < 0.001
NHS website	100.0 (36/36) [90, 100]	24.3 (9/37) [13, 40]	+75.7	3.95 (2.27, 6.85), *q* < 0.001
Lullaby Trust	63.9 (23/36) [47, 78]	0.0 (0/37) [0, 9]	+63.9	48.27 (3.04, 766.00), *q* < 0.001
Breastfeeding support before birth	100.0 (36/36) [90, 100]	10.8 (4/37) [4, 25]	+89.2	8.33 (3.50, 19.85), *q* < 0.001
Breastfeeding support after birth	100.0 (36/36) [90, 100]	24.3 (9/37) [13, 40]	+75.7	3.95 (2.27, 6.85), *q* < 0.001

Prevalence-ratio reference category is Experienced. Ethnicity and parity are completely confounded in the usable sample. All 36 first-time parents identified as White British, and all 28 other respondents identified as experienced parents. Estimates here should not be read as parity effects independent of ethnicity. All items are self-reported. Wilson 95 percent confidence intervals are shown. Prevalence ratios are Haldane-corrected and are calculated as First-time divided by Experienced. pp, percentage points. *q* is the Benjamini–Hochberg adjusted *p*-value, applied separately across the 14 parity comparisons.

### Composite scale results

The eight knowledge items yielded a Kuder-Richardson 20 coefficient of 0.94. The six engagement items yielded 0.93. Both values indicate high internal consistency for the binary item sets (19). Internal consistency does not by itself establish construct validity, and the two composite indices should be interpreted as descriptive summaries. The Knowledge Proportion Score and the Engagement Proportion Score were very strongly correlated overall (Spearman's *ρ* = 0.965, *p* < 0.001). White British respondents had a median KPS of 87.5 (interquartile range 50.0–100.0). The Other group had a median KPS of 0.0 (IQR 0.0–0.0). Median EPS values followed the same separation. Mann–Whitney *U* tests gave *p*-values in the approximate range of 1.0 × 10^−12^ to 8.3 × 10^−14^ across the composite-score comparisons by ethnicity and parity. The composite-score summary is presented in Table 4. Figure 2 plots KPS against EPS across the three joint ethnicity-parity profiles present in the sample. The pooled association reflects both marked between-profile separation and substantial within-profile co-variation. Within-profile Spearman correlations were 0.831 for White British/First-time respondents, 0.879 for White British/Experienced respondents, and 0.651 for Other/Experienced respondents (Supplementary Table S5). Sensitivity analysis recoding Unsure as missing produced similar reliability coefficients and group-comparison conclusions (Supplementary Table S4).

**Table 4:** Composite score reliability, association, and group comparisons.

Metric	Estimate	Test statistic, *p*
Internal consistency (KR-20), Knowledge (8 items)	0.94	Not applicable
Internal consistency (KR-20), Engagement (6 items)	0.93	Not applicable
Association KPS with EPS (pooled), Spearman's *ρ*	0.965	*p* < 0.001
KPS by ethnicity, White British vs. Other	Median 87.5 (IQR 50.0–100.0) vs. 0.0 (IQR 0.0–0.0)	Mann–Whitney *U*, *p* ≈ 1.0 × 10^−12^
EPS by ethnicity, White British vs. Other	Median 83.3 (IQR 66.7–100.0) vs. 0.0 (IQR 0.0–0.0)	Mann–Whitney *U*, *p* ≈ 1.5 × 10^−12^
KPS by parity, First-time vs. Experienced	Median 87.5 (IQR 62.5–100.0) vs. 0.0 (IQR 0.0–25.0)	Mann–Whitney *U*, *p* ≈ 1.2 × 10^−13^
EPS by parity, First-time vs. Experienced	Median 91.7 (IQR 66.7–100.0) vs. 0.0 (IQR 0.0–16.7)	Mann–Whitney *U*, *p* ≈ 8.3 × 10^−14^

KPS, Knowledge Proportion Score; EPS, Engagement Proportion Score; IQR, interquartile range. High KR-20 values indicate internal consistency and should not be interpreted alone as evidence of validated psychometric scales. Composite scores were computed only for respondents with at least half of the relevant items answered.

**Figure 2:**
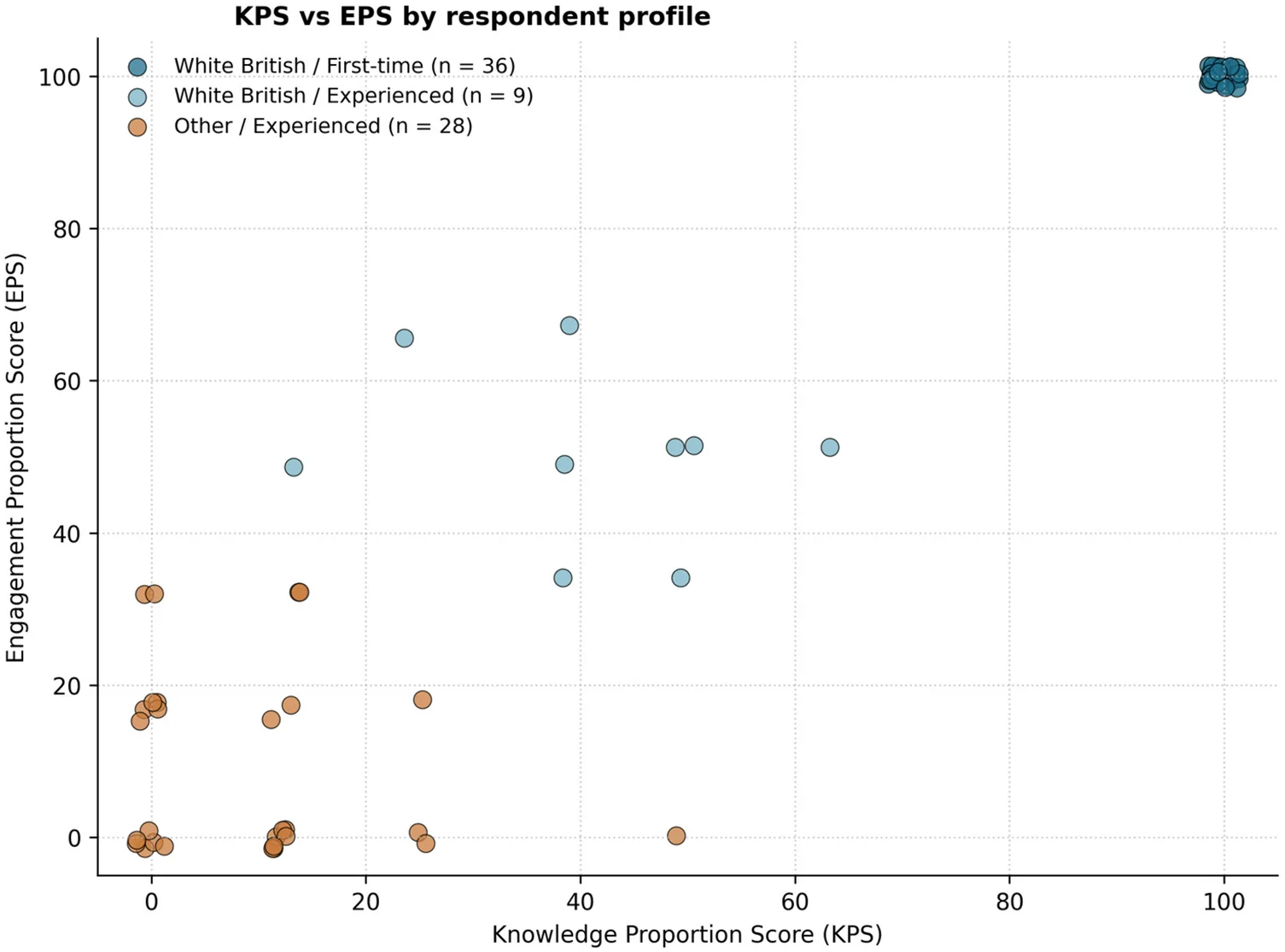
Scatter of the knowledge proportion score (KPS) against the engagement proportion score (EPS) at the respondent level, coloured by the joint ethnicity-parity profile. Three joint profiles are present in the sample (White British/First-time, White British/Experienced, and Other/Experienced). The pooled association reflects both separation between these profiles and positive within-profile co-variation between KPS and EPS.

## Discussion

Birth partners and co-parents in this single-site exploratory sample reported variable exposure to infant safety information. Reported exposure was highest for smoking risk, breastfeeding information, and safe sleep, and lowest for practical emergency-care topics such as bystander life support, choking response, and first aid. Engagement items followed a related pattern. NHS website use and breastfeeding support were more frequently reported than antenatal class attendance or awareness of dedicated safe-sleep resources such as the Lullaby Trust. These findings suggest that partners in this setting were more frequently exposed to general health-promotion messaging than to practical acute-care skills relevant to the immediate postnatal period. The low reported exposure to bystander life support, choking response, and first aid is the most consistent descriptive finding across topics, respondent profiles, and recruitment period, and is the finding that appears most resilient to the limitations discussed below.

Substantial differences were observed between the two contrasting respondent profiles. Birth partners identified as White British and First-time reported markedly higher exposure to information and higher engagement with services than respondents in the combined Other and Experienced profile. These contrasts appeared at the item level and in the composite scores. They are best read as descriptive comparisons between two respondent profiles that happen to differ on several correlated attributes, not as estimates of an ethnic effect or a parity effect in isolation. Ethnicity and parity are completely confounded in the usable dataset, so the analyses in Tables 2, 3 cannot separate their contributions. In addition, the study did not collect information on educational attainment, occupation, area-level deprivation, migration history, English language proficiency, digital access, or health literacy, any of which could plausibly account for part of the observed variation. These contrasts should not be characterised as ethnic inequities on the basis of the present design.

The composite-score findings help characterise the joint structure of the responses. Both item sets showed high internal consistency, and the two indices were strongly correlated in the pooled sample. Figure 2 and Supplementary Table S5 indicate that this coefficient reflects two components, substantial separation between the joint respondent profiles and appreciable positive co-variation within each of the three profiles present. The within-profile coefficients ranged from 0.651 to 0.879, so the pooled association should not be attributed solely, or mainly, to between-profile separation. A shared information environment is one possible explanation, but the cross-sectional design cannot establish temporal or causal direction. Partners who feel more confident may be more likely to seek information and service contact, whereas greater contact may also increase reported exposure. This dataset cannot distinguish those explanations.

The findings are broadly consistent with wider literature on the evolving role of birth partners and fathers in perinatal and neonatal care (1, 2, 6). Contemporary UK and international guidance places partners within everyday caregiving, including involvement in decision-making, practical newborn care, and support for maternal recovery after birth (2, 17). Intervention studies suggest that partner-inclusive programmes may improve maternal and infant outcomes. Programmes designed for fathers have been associated with increased rates of exclusive breastfeeding, with reported relative risks in the range 1.28–1.35 (3). Partner involvement has also been associated with improved maternal mental health outcomes, although effect magnitude and persistence vary across settings (4). No intervention was evaluated in the present descriptive study.

The wider literature also indicates that partners often experience maternity care environments as primarily oriented toward mothers and describe information resources that are not designed with them in mind (1, 2, 6). Partners from ethnic minority backgrounds may be less frequently included in clinical conversations about infant care and safety (1, 5, 16). Earlier work has raised concerns that perinatal education in the United Kingdom may draw implicitly on assumptions about White middle-class family structures, which may limit accessibility for more diverse populations (5). The descriptive pattern observed here is broadly consistent with those concerns. It is not, on its own, evidence for them. The design cannot separate the contribution of ethnicity from parity, socioeconomic circumstance, language, digital access, or recruitment reach.

### What this study adds

Within the design's limits, this study provides four contributions. First, it offers descriptive data on birth partners' and co-parents' self-reported exposure to eight infant safety topics in one large UK maternity service. Second, it identifies low reported exposure to practical emergency-care topics, namely bystander life support, choking response, and first aid, across respondent profiles. Third, it suggests that self-reported exposure to information and self-reported engagement with services may be unevenly distributed across contrasting respondent profiles in this setting, although the confounded design does not allow the drivers of that variation to be identified. Fourth, it generates hypotheses about the reach of partner-focused perinatal education that should be tested in larger multi-site studies with sufficient statistical power to separate ethnicity, parity, socioeconomic position, and language.

### Strengths and limitations

A strength of the study is the collection of primary data on a group under-represented in perinatal survey research, namely birth partners and co-parents themselves, in a service with a substantial minority ethnic population in its catchment. A second strength is the explicit examination of self-reported knowledge and self-reported engagement together, with the internal consistency of the two composite indices estimated and reported. The limitations are substantive and should be read as constraints on interpretation. First, the sample is small (*n* = 73) and drawn from a single UK teaching hospital by convenience sampling on weekday clinic and ward attendances. A record of eligibility, approach, and refusal was not systematically maintained, so a formal response rate cannot be reported. Partners with insufficient English proficiency were not enrolled, since translated materials and interpreter services were not available. This design will under-represent partners facing the greatest linguistic and structural barriers to service engagement, who may also carry the greatest need for targeted safety information.

Second, ethnicity and parity are completely confounded in the usable dataset, which prevents statistical separation of their contributions. Third, the Other ethnicity category combines respondents self-describing as Romanian, Kurdish, Chinese, African, Indian, Pakistani, Bengali, Albanian, Nigerian, and Polish. This heterogeneous grouping was analytically necessary at *n* = 28, but it obscures within-category variation in migration experience, language, socioeconomic position, cultural background, and access to services. Fourth, information on educational attainment, occupation, area-level deprivation, migration history, English language proficiency, digital access, and health literacy was not collected. Any of these variables could plausibly account for part of the observed variation between respondent profiles.

Fifth, all measures are self-reported. Partners described whether they had received information and whether they felt they knew what to do. These self-reports may not reflect actual knowledge or behaviour in an emergency. Preparedness is often over-stated in socially sensitive domains, so recall bias and social desirability bias are both plausible. Objective testing of knowledge and observation of behaviour were not undertaken. Sixth, the recruitment window sampled both antenatal and postnatal respondents. Several items are intrinsically time-conditional in the perinatal pathway, including post-birth breastfeeding support. Stratified item-level results in Supplementary Table S2 show no recruitment-period comparison surviving multiplicity correction, although the modest sample limits the precision of this assessment and formal multivariable adjustment was not feasible. The antenatal-class item also combined actual attendance with intention to attend. Those are distinct behaviours and cannot be separated retrospectively from the item as fielded, so the result should not be interpreted as a measure of completed class attendance. Seventh, the composite indices were derived for this study and have not been externally validated. Their equal item weighting is pragmatic and does not imply equivalent clinical importance across domains. High internal consistency does not establish construct validity, and the KPS and EPS should be read as descriptive summaries rather than validated latent-construct measures. Eighth, the use of Haldane-corrected prevalence ratios produced very wide confidence intervals in the presence of zero cells, which the reader should weigh when interpreting the point estimates in Tables 2, 3. Absolute proportions and absolute differences between profiles are more stable and are reported alongside the ratios.

Ninth, the cross-sectional design precludes causal inference regarding the KPS and EPS association. Tenth, no infant outcome measure, behavioural measure, or observed competence measure was collected. The pathway from self-reported partner exposure to reduced infant mortality risk is clinically plausible but is not tested by the present design. Finally, the study reflects the experience of partners who were present in the clinic or ward on the days approached and willing to complete a written questionnaire in English. Findings may not transfer to settings with different models of antenatal education, different parental leave arrangements, or different norms of partner involvement.

## Conclusion

In this exploratory single-site sample of 73 birth partners and co-parents, self-reported exposure to infant safety information varied across topics. Smoking risk, breastfeeding information, safe sleep, and NHS website use were most commonly reported. Exposure to information about choking response, basic life support, and first aid was less common across respondent profiles. Substantial differences were observed between two contrasting respondent profiles defined by ethnicity and parity, although the two demographic axes are completely confounded in the usable sample and the study did not collect socioeconomic, language, or health-literacy information. The design does not support causal claims about ethnic inequities or about infant mortality risk reduction. Within these limits, the findings identify practical emergency-care topics as a candidate priority for reinforcement in partner-facing perinatal education, and they support the case for larger multi-site studies designed to separate the contributions of ethnicity, parity, and other social determinants. The findings should be read as hypothesis-generating.

## Data Availability

The original contributions presented in the study are included in the article/Supplementary Material, further inquiries can be directed to the corresponding author.

## Supplementary material

Supplementary Material A Full python analytical script.

Supplementary Table S1 Participant characteristics stratified by recruitment period, including ethnicity, parity, and age group.

Supplementary Table S2 Item-level yes proportions with Wilson 95 percent confidence intervals, stratified by recruitment period.

Supplementary Table S3 Raw distribution of yes, no, unsure, and missing responses for every knowledge and engagement item.

Supplementary Table S4 Sensitivity analysis 1, item-level results with unsure recoded as missing.

Supplementary Table S5 Within-profile Spearman correlations between KPS and EPS.
