# Supplementary material for: Reported infant safety information and perinatal service engagement among birth partners and co-parents. An exploratory cross-sectional survey at a large UK teaching hospital
Source: Front Pediatr. 2026 Oct 13;14:1884421. doi: 10.3389/fped.2026.1884421 (PMC13710227; doi:10.3389/fped.2026.1884421)
Supplement: Supplementary file 1 [file Supplementaryfile1.docx]

**Supplementary Materials**

*Reported Infant Safety Information and Perinatal Service Engagement Among Birth Partners and Co-Parents. An Exploratory Cross-Sectional Survey at a Large UK Teaching Hospital*

This document collects the supplementary tables and figures referenced in the manuscript. Supplementary Material A (the full Python analytical script) is provided as a separate file. Supplementary Tables S1 through S5 and both figures follow.

## **Supplementary Table S1. Participant characteristics stratified by recruitment period**

Panel A. Recruitment period by ethnicity (n = 73).

| **Recruitment period** | **White British** | **Other** | **Total** |
| --- | --- | --- | --- |
| Antenatal | 25 | 15 | 40 |
| Postnatal | 20 | 13 | 33 |
| **Total** | **45** | **28** | **73** |

***Note.*** *Joint counts computed from the study CSV by analysis_code.py.*

Panel B. Recruitment period by parity (n = 73).

| **Recruitment period** | **First-time** | **Experienced** | **Total** |
| --- | --- | --- | --- |
| Antenatal | 18 | 22 | 40 |
| Postnatal | 18 | 15 | 33 |
| **Total** | **36** | **37** | **73** |

Panel C. Recruitment period by age group (n = 73).

| **Recruitment period** | **16 to 21** | **22 to 30** | **31 to 40** | **Over 40** | **Total** |
| --- | --- | --- | --- | --- | --- |
| Antenatal | 1 | 11 | 23 | 5 | 40 |
| Postnatal | 1 | 13 | 18 | 1 | 33 |
| Total | 2 | 24 | 41 | 6 | 73 |

## **Supplementary Table S2. Item-level Yes proportions with Wilson 95 percent confidence intervals, stratified by recruitment period**

| **Item** | **Antenatal (n = 40) Yes n/N (%) [95% CI]** | **Postnatal (n = 33) Yes n/N (%) [95% CI]** | Fisher p | BH q |
| --- | --- | --- | --- | --- |
| ***Knowledge or awareness (self-reported)*** |  |  |  |  |
| Benefits of breastfeeding | 20/40 (50.0) [35.2, 64.8] | 23/33 (69.7) [52.7, 82.6] | 0.101 | 0.353 |
| Basic (bystander) life support | 6/40 (15.0) [7.1, 29.1] | 7/33 (21.2) [10.7, 37.8] | 0.549 | 0.980 |
| Dealing with choking | 14/40 (35.0) [22.1, 50.5] | 9/33 (27.3) [15.1, 44.2] | 0.614 | 0.980 |
| Recognising signs of illness | 20/40 (50.0) [35.2, 64.8] | 15/33 (45.5) [29.8, 62.0] | 0.815 | 1.000 |
| Risks of smoking | 28/40 (70.0) [54.6, 81.9] | 23/33 (69.7) [52.7, 82.6] | 1.000 | 1.000 |
| Risks of shaking / crying baby | 17/40 (42.5) [28.5, 57.8] | 21/33 (63.6) [46.6, 77.8] | 0.1000 | 0.353 |
| Safe sleep practices | 24/40 (60.0) [44.6, 73.7] | 19/33 (57.6) [40.8, 72.8] | 1.000 | 1.000 |
| First aid | 14/39 (35.9) [22.7, 51.6] | 11/33 (33.3) [19.8, 50.4] | 1.000 | 1.000 |
| ***Engagement (self-reported)*** |  |  |  |  |
| Mental health support | 18/40 (45.0) [30.7, 60.2] | 22/33 (66.7) [49.6, 80.2] | 0.0977 | 0.353 |
| Attended or intended antenatal classes | 7/40 (17.5) [8.7, 32.0] | 11/33 (33.3) [19.8, 50.4] | 0.173 | 0.483 |
| NHS website awareness | 26/40 (65.0) [49.5, 77.9] | 19/33 (57.6) [40.8, 72.8] | 0.630 | 0.980 |
| Lullaby Trust support awareness | 12/40 (30.0) [18.1, 45.4] | 11/33 (33.3) [19.8, 50.4] | 0.804 | 1.000 |
| Breastfeeding support before birth | 19/40 (47.5) [32.9, 62.5] | 21/33 (63.6) [46.6, 77.8] | 0.238 | 0.555 |
| Breastfeeding support after birth | 20/40 (50.0) [35.2, 64.8] | 25/33 (75.8) [59.0, 87.2] | 0.0308 | 0.353 |

***Note. Values use the primary coding (Unsure recoded as No). Wilson 95 percent confidence intervals are shown. First aid Antenatal n = 39 because of one unanswered response. Fisher's exact tests compare antenatal with postnatal respondents for each item, and BH q-values apply the Benjamini-Hochberg correction across the 14 recruitment-period tests. No item-level comparison remains statistically significant after correction; the smallest raw p-value is for post-birth breastfeeding support (p = 0.0308, q = 0.353). KPS and EPS also show no statistical evidence of a recruitment-period difference (Mann-Whitney p = 0.730 and p = 0.204). The descriptive contrast for post-birth breastfeeding support remains time-conditional because antenatal respondents may not yet have had an opportunity to receive post-birth support.***

## **Supplementary Table S3. Raw distribution of Yes, No, Unsure, and missing responses for every knowledge and engagement item (n = 73)**

| **Item** | **Yes** | **No** | **Unsure** | **Missing** | **Total** |
| --- | --- | --- | --- | --- | --- |
| ***Knowledge or awareness (self-reported)*** |  |  |  |  |  |
| Benefits of breastfeeding | 43 | 28 | 2 | 0 | 73 |
| Basic (bystander) life support | 13 | 53 | 7 | 0 | 73 |
| Dealing with choking | 23 | 44 | 6 | 0 | 73 |
| Recognising signs of illness | 35 | 30 | 8 | 0 | 73 |
| Risks of smoking | 51 | 19 | 3 | 0 | 73 |
| Risks of shaking / crying baby | 38 | 31 | 4 | 0 | 73 |
| Safe sleep practices | 43 | 26 | 4 | 0 | 73 |
| First aid | 25 | 44 | 3 | 1 | 73 |
| ***Engagement (self-reported)*** |  |  |  |  |  |
| Mental health support | 40 | 29 | 4 | 0 | 73 |
| Attended or intended antenatal classes | 18 | 55 | 0 | 0 | 73 |
| NHS website awareness | 45 | 27 | 1 | 0 | 73 |
| Lullaby Trust support awareness | 23 | 49 | 1 | 0 | 73 |
| Breastfeeding support before birth | 40 | 31 | 2 | 0 | 73 |
| Breastfeeding support after birth | 45 | 24 | 4 | 0 | 73 |

***Note.*** *Yes counts match the item-level totals in Table 1b of the main manuscript. Missing refers to genuinely unanswered items; only first aid had any missing responses (1 of 73, 1.4 percent). Unsure responses were treated as No in the primary analysis and as missing in the sensitivity analysis (Table S4). Antenatal classes was fielded without an Unsure option in the questionnaire, so all 73 respondents answered Yes or No.*

## **Supplementary Table S4. Sensitivity analysis, item-level results with Unsure recoded as missing rather than as No**

This table repeats the primary item-level analyses (Tables 1b, 2, and 3 in the main manuscript) with Unsure responses recoded as missing and excluded from denominators. The primary analysis (Unsure recoded as No) is retained in the main manuscript on the safety-critical rationale set out in the Methods.

| **Item** | **Overall Yes/N (%) [95% CI]** | **White British vs Other** | **First-time vs Experienced** | **q (eth / par)** |
| --- | --- | --- | --- | --- |
| ***Knowledge or awareness (self-reported)*** |  |  |  |  |
| Benefits of breastfeeding | 43/71 (60.6) [48.9, 71.1] | WB 40/45 (88.9), Other 3/26 (11.5), PR 7.70 (2.64, 22.44) | FT 36/36 (100.0), Exp 7/35 (20.0), PR 4.74 (2.50, 8.96) | <0.001 / <0.001 |
| Basic (bystander) life support | 13/66 (19.7) [11.9, 30.8] | WB 13/45 (28.9), Other 0/21 (0.0), PR 12.91 (0.80, 207.44) | FT 13/36 (36.1), Exp 0/30 (0.0), PR 22.62 (1.40, 365.40) | 0.006 / <0.001 |
| Dealing with choking | 23/67 (34.3) [24.1, 46.3] | WB 23/45 (51.1), Other 0/22 (0.0), PR 23.50 (1.49, 369.85) | FT 23/36 (63.9), Exp 0/31 (0.0), PR 40.65 (2.57, 642.85) | <0.001 / <0.001 |
| Recognising signs of illness | 35/65 (53.8) [41.9, 65.4] | WB 35/45 (77.8), Other 0/20 (0.0), PR 32.41 (2.09, 503.57) | FT 33/36 (91.7), Exp 2/29 (6.9), PR 13.29 (3.48, 50.81) | <0.001 / <0.001 |
| Risks of smoking | 51/70 (72.9) [61.5, 81.9] | WB 45/45 (100.0), Other 6/25 (24.0), PR 3.96 (2.03, 7.70) | FT 36/36 (100.0), Exp 15/34 (44.1), PR 2.23 (1.53, 3.24) | <0.001 / <0.001 |
| Risks of shaking / crying baby | 38/69 (55.1) [43.4, 66.2] | WB 37/45 (82.2), Other 1/24 (4.2), PR 19.73 (2.88, 135.08) | FT 35/36 (97.2), Exp 3/33 (9.1), PR 10.69 (3.63, 31.50) | <0.001 / <0.001 |
| Safe sleep practices | 43/69 (62.3) [50.5, 72.8] | WB 43/45 (95.6), Other 0/24 (0.0), PR 47.28 (3.04, 735.84) | FT 36/36 (100.0), Exp 7/33 (21.2), PR 4.47 (2.37, 8.42) | <0.001 / <0.001 |
| First aid | 25/69 (36.2) [25.9, 48.0] | WB 25/45 (55.6), Other 0/24 (0.0), PR 27.72 (1.76, 436.27) | FT 25/36 (69.4), Exp 0/33 (0.0), PR 46.86 (2.97, 740.38) | <0.001 / <0.001 |
| ***Engagement (self-reported)*** |  |  |  |  |
| Mental health support | 40/69 (58.0) [46.2, 68.9] | WB 38/45 (84.4), Other 2/24 (8.3), PR 10.13 (2.67, 38.42) | FT 36/36 (100.0), Exp 4/33 (12.1), PR 7.45 (3.15, 17.64) | <0.001 / <0.001 |
| Attended or intended antenatal classes | 18/73 (24.7) [16.2, 35.6] | WB 18/45 (40.0), Other 0/28 (0.0), PR 23.33 (1.46, 372.39) | FT 18/36 (50.0), Exp 0/37 (0.0), PR 38.00 (2.38, 607.86) | <0.001 / <0.001 |
| NHS website awareness | 45/72 (62.5) [51.0, 72.8] | WB 44/45 (97.8), Other 1/27 (3.7), PR 26.40 (3.86, 180.77) | FT 36/36 (100.0), Exp 9/36 (25.0), PR 3.84 (2.22, 6.66) | <0.001 / <0.001 |
| Lullaby Trust support awareness | 23/72 (31.9) [22.3, 43.4] | WB 23/45 (51.1), Other 0/27 (0.0), PR 28.61 (1.81, 452.70) | FT 23/36 (63.9), Exp 0/36 (0.0), PR 47.00 (2.96, 745.48) | <0.001 / <0.001 |
| Breastfeeding support before birth | 40/71 (56.3) [44.8, 67.3] | WB 39/45 (86.7), Other 1/26 (3.8), PR 22.53 (3.29, 154.52) | FT 36/36 (100.0), Exp 4/35 (11.4), PR 7.89 (3.32, 18.75) | <0.001 / <0.001 |
| Breastfeeding support after birth | 45/69 (65.2) [53.4, 75.4] | WB 40/45 (88.9), Other 5/24 (20.8), PR 4.27 (1.94, 9.37) | FT 36/36 (100.0), Exp 9/33 (27.3), PR 3.53 (2.06, 6.07) | <0.001 / <0.001 |

***Note. Under this sensitivity coding, Kuder-Richardson 20 reliability was 0.927 for the knowledge item set and 0.917 for the engagement item set (compared with 0.94 and 0.93 respectively under the primary coding). PR = Haldane-corrected prevalence ratio. The ratios are calculated as White British divided by Other and First-time divided by Experienced, so the corresponding reference categories are Other and Experienced. q is the Benjamini-Hochberg adjusted p-value, applied separately within each 14-test demographic comparison. The substantive direction and magnitude of the primary findings are consistent between the two codings.***

## **Supplementary Table S5. Spearman rank correlations between the Knowledge Proportion Score and the Engagement Proportion Score, pooled and within respondent profile**

| **Group** | **Spearman ρ** | **p-value** |
| --- | --- | --- |
| Pooled sample (n = 73) | 0.965 | 5.82e-43 |
| Within White British / First-time (n = 36) | 0.831 | 3.57e-10 |
| Within White British / Experienced (n = 9) | 0.879 | 0.00182 |
| Within Other / Experienced (n = 28) | 0.651 | 0.000177 |

***Note. The pooled Spearman correlation of 0.965 is reported in the main manuscript. Within-profile correlations remain strong across all three joint profiles (0.651 to 0.879), although they are lower than the pooled value. Figure 2 indicates that the pooled coefficient reflects both marked separation between the three respondent profiles and substantial within-profile co-variation between KPS and EPS.***

# **Supplementary Figures**

Both figures are reproduced from the main manuscript. Higher-resolution 300 DPI PNG files are supplied alongside this document as Figure_1.png and Figure_2.png.

**Figure 1.** Combined item-level self-reported frequencies for the eight knowledge items and six engagement items reported by 73 birth partners and co-parents. Yes responses are shown as percentages on the horizontal axis, ranked from highest to lowest. Whiskers indicate Wilson 95 percent confidence intervals.

**Figure 2. Scatter of the Knowledge Proportion Score (KPS) against the Engagement Proportion Score (EPS) at the respondent level, coloured by the joint ethnicity-parity profile. Three profiles are present in the sample. The pooled association reflects both between-profile separation and positive within-profile co-variation.**
